# Molecular Tilting Alignment on Ag@C Nanocubes Monitored by Temperature-Dependent Surface Enhanced Raman Scattering

**DOI:** 10.1038/s41598-017-13022-x

**Published:** 2017-10-16

**Authors:** Yinong Wang, Yinghui Sun, Di Chen, Xiaofang Zhang, Lin Guo, Rongming Wang

**Affiliations:** 1 Department of Physics, Beihang University, Beijing, 100191 China; 2 Beijing Key Laboratory for Magneto-Photoelectrical Composite and Interface Science, School of Mathematics and Physics, University of Science and Technology Beijing, Beijing, 100083 China; 3 School of Chemistry and Environment, Beihang University, Beijing, 100191 China

## Abstract

Core@shell Ag@C nanocubes (NCs) with a cubic silver core (~60 nm of side length) and a coating of ultrathin amorphous carbon (~4 nm) have been synthesized on a large scale by a one-pot hydrothermal method. The carbon layer not only protects the Ag@C nanocubes from oxidation under hydrothermal condition, but also stabilizes the structure of Ag cores. Considering that optical properties of nanostructured metals strongly depend on the temperature for SERS measurement, in this work we systemically investigate the relationship between the orientation of molecules adsorbed on Ag@C NCs and temperature by SERS spectra. Results suggest that the adsorbed 4-MBA molecules prefer a flat orientation on the NC surface with temperature decreasing. In addition, Ag@C NCs after one-year storage in water still maintain high SERS-active capability. Our synthesized Ag@C NCs with excellent and stable optical properties can be potentially applied in the field of sensor and ultrasensitive spectral analysis.

## Introduction

Noble metals, including Au, Ag, and Cu, have attracted significant attention due to their extraordinary optical properties and potential applications in a series of technologies, such as bio-molecular sensing^1–4^, molecule trace detection^5–7^, and surface plasmon-driven chemical reactions^8,9^. These applications are based on the surface plasmon resonance (SPR) effect of metals^10,11^, which is attributed to the mechanism of electromagnetic enhancement. The SPR effect can be utilized in the analytic technique of surface enhanced Raman scattering (SERS)^12^. It was found that the SERS effect was extremely sensitive to the environment of noble metal substrate^13,14^.

Ag is known as the best metal for SERS substrate with enhancement factors greater than 10^8^, and has been widely applied in Raman detection since the discovery of SERS in 1970s^3^. Nie *et al*. have reported that the ‘hot spot’ is responsible for the SERS enhancement^6^. Theoretical calculations of SERS property of Ag nanoparticles with various shapes^15–23^ have reached fairly high level of maturity^24,25^. According to theoretical models, the electromagnetic enhancement factor can be 5 × 10^4^ for hot spot enhancement^26^ by varying the curvature and the shape of nanoparticles^27,28^. Although Ag SERS substrates have been widely studied, it is still challenging to extend the applications of SERS to detect analytes. First, it is difficult to maintain Ag optical properties under real working conditions in Raman experiment^29,30^. Second, the chemical bond between a molecule and the substrate also influences the measure results of SERS. Ag nanocrystals like colloidal and nanorods with carbon coatings had been reported^24,31,32^, and their stability in vacuum was studied. Li *et al*. reported Ag@C core-shell structured composite nanoparticles, which were obtained by a green wet chemical method using water as solvent and glucose as reducing agent^33,34^. These sizes and shapes of their Ag nanoparticles were not uniform, and the carbon coatings over Ag nanoparticles were tens of nm in thickness.

In our work, we developed a hydrothermal method to grow Ag@C NCs with very narrow size distribution and high property-stability in water, which show extraordinary electromagnetic field due to the antenna effect. The merit of our method is that the crystal growth is well controlled, leading to cubic Ag crystals with uniform size. Also, carbon shells over Ag NCs were one-pot synthesized, following the completion of Ag NCs growth. The structure and optical properties of Ag@C NCs were characterized using SEM (scanning electron microscope), TEM (transmission electron microscopy), EDS (energy-dispersive X-ray spectrometer), UV-VIS-IR (ultraviolet-visible-infrared) absorption spectrum, XPS (electron diffraction and X-ray photoelectron spectroscopy, X-ray source, 1486.6 eV) and Raman spectra (488 nm), respectively. The temperature-dependent orientation of molecules adsorbed onto Ag@C NCs was revealed by means of changing temperatures from 10 K to 190 K in SERS measurement. The stability of Ag@C NCs in the water solution has been investigated by analyzing the sample after storage in water for 12 months. High SERS activity of Ag@C NCs was well maintained with aging.

## Results Section

### Electron Microscopy Observation and Elemental Composition Analysis

**Figure 1:**
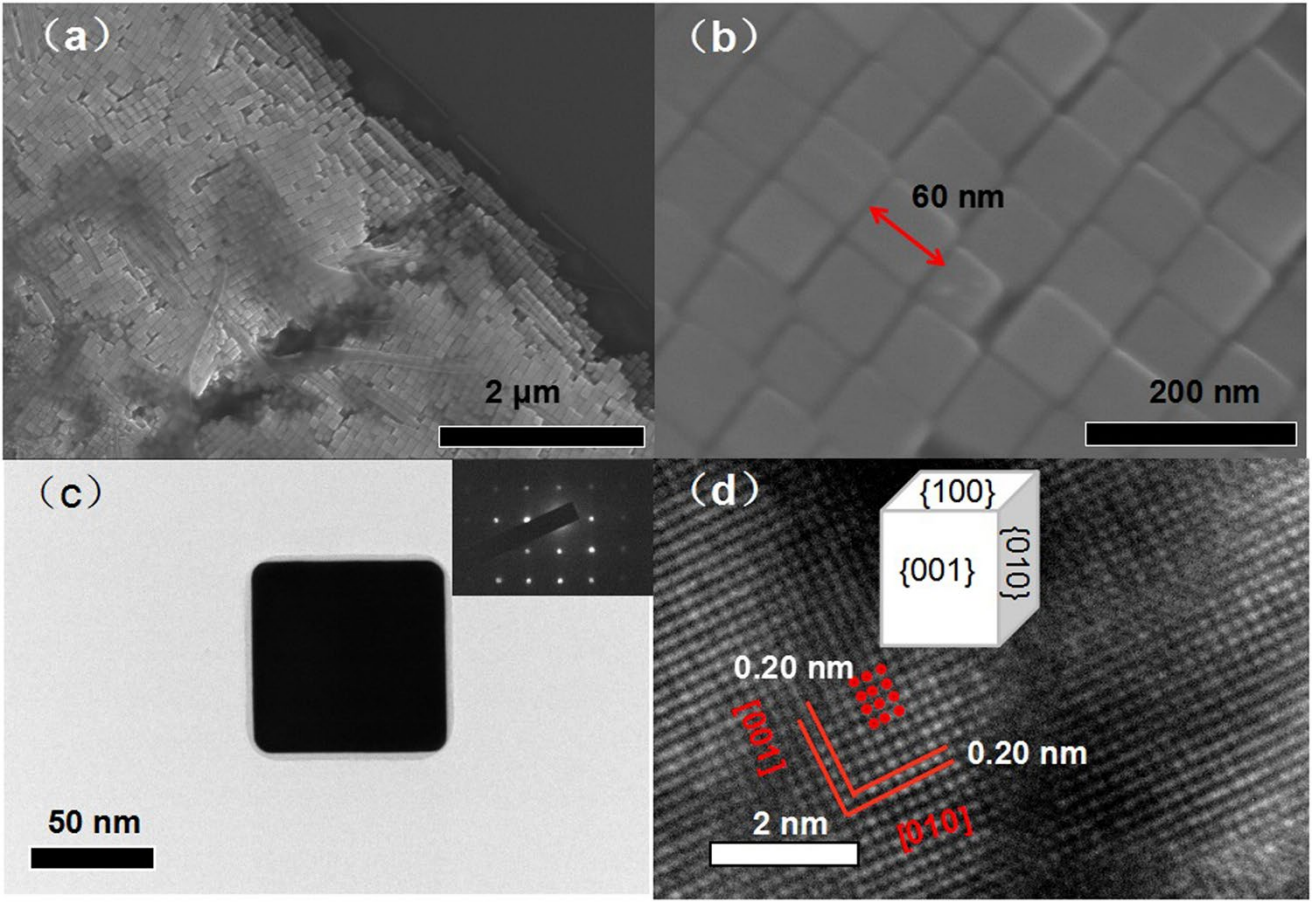
(**a**) and (**b**) SEM images of Ag@C NCs dispersed onto Si substrate. (**c**) TEM image of a single Ag@C nanocube. The inset is the corresponding SAED. (**d**) HR-TEM image of Ag@C NC.

A typical low-magnification SEM image in Fig. 1a shows that the sample contains densely-packed NCs with uniform size. The EDS data (Figure S1) shows that the main signal is ascribed to Ag element with atomic percentage up to 95%. The side length of Ag@C NC is about 60 nm as shown in Fig. 1b. The TEM image in Fig. 1c clearly shows the core-shell structure, i.e., Ag cube coated with a 4-nm-thick carbon layer. The corresponding SAED pattern obtained from the [100] axis presents a perfect square shape shown in the inset of Fig. 1c, demonstrating its single crystal structure. The lattice fringe of this single-crystal Ag cube is shown in the HRTEM image in Fig. 1d. The lattice inter-spacing is measured to be 0.20 nm, corresponding to (001) plane of the f.c.c. Ag crystal. More TEM images at different magnifications can be found in Figure S2.

**Figure 2:**
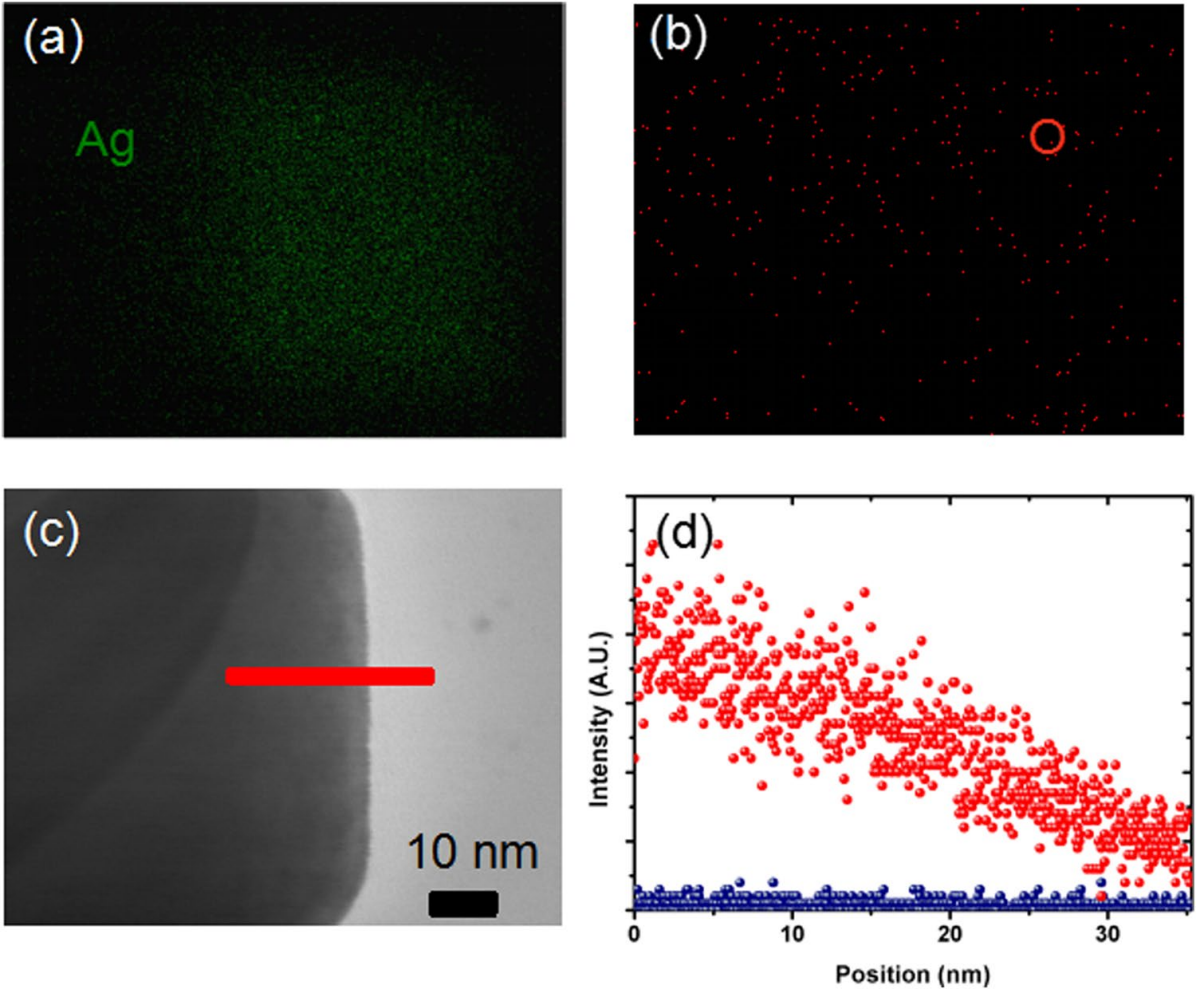
(**a**) STEM-EDS mapping of AgK in the Ag@C NC. (**b**) STEM-EDS of OK in the Ag@C NC. (**c**) STEM image of the Ag@C NC edge (**d**) the line STEM-EDS of AgK and OK in the Ag@C NC.

Figures 2a and b display the scanning TEM-energy-dispersive X-ray spectroscopy (STEM-EDS) elemental mapping images of the NCs, which illustrate the Ag and O distribution in Ag@C NCs, respectively. The blurred green square in Fig. 2a indicates the distribution of Ag element. Except this, oxygen element, of which the contend given by EDS is 4.2 at.%, is barely detected in Fig. 2b. The C element mapping image is shown in Figure S3, which confirms the carbon coating on Ag@C NC. Figure 2c represents the STEM image of the NC edge, along the red line the distribution of Ag and O element is recorded, as shown in Fig. 2d. It further demonstrates the high purity of our Ag@C NCs. Oxidation is prohibited during the hydrothermal process because of the protection of carbon coating. The weak O element signal in Fig. 2d may come from the carbon supported membrane substrate. Point STEM-EDS images (Figure S1,) further support that these NCs contain a single element of silver.

### XPS analysis

**Figure 3:**
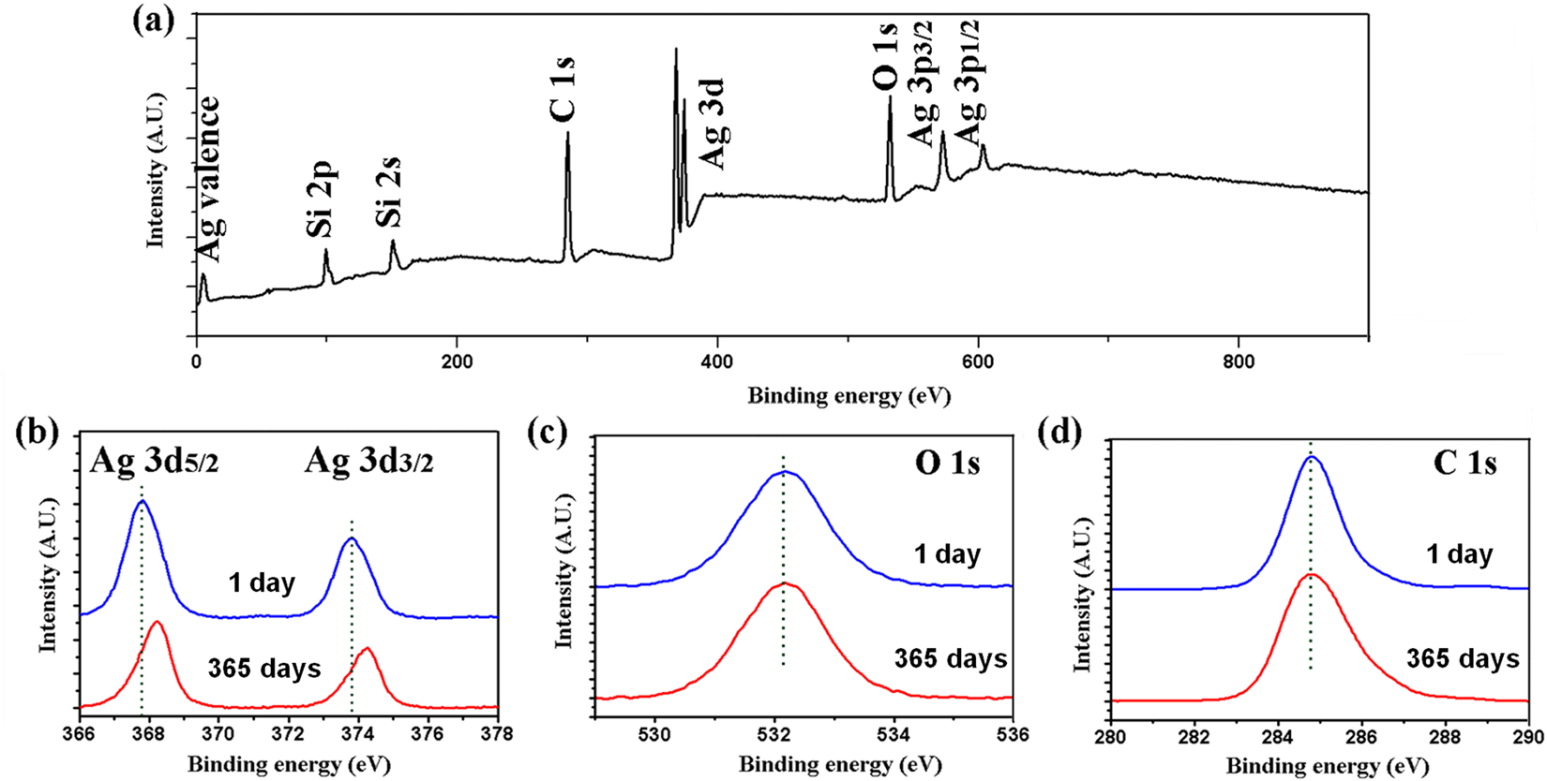
(**a**) Survey XPS spectrum of Ag NCs, (**b**) Ag3d, (**c**) O1s and (**d**) C1s fine XPS spectra of Ag NCs. The blue curves are obtained from the fresh samples and the red curves are from the aged one.

A pile of densely-packed Ag@C NCs on Si substrate was studied by using XPS. Figure 3a displays the XPS spectrum of as-prepared Ag NCs. Except two weak peaks assigned to Si, all other strong peaks are contributed by Ag or C. Li *et al*. have synthesized similar Ag@C core-shell nanoparticles through the reduction of Ag^+^ under hydrothermal environment^31^. In that experiment, two synthesis steps have been considered in the process: (1) Growing Ag nanoparticles with the restraint of CTAB (Hexadecyl trimethyl ammonium Bromide); (2) Carbonization with CTAB and the formation of amorphous carbon layer on the Ag NCs. The schematic illustration is shown in Figure S4. To further investigate the stability of our Ag@C NCs prepared by a hydrothermal method, we measured XPS on the sample the next day after synthesis and after 365 days storage in water, respectively. Figure 3b shows the Ag 3d fine XPS spectra of Ag@C NCs 1 day and 365 days after synthesis. For the freshly-prepared sample, two peaks in the red-line curve at 368.1 eV and 374.1 eV are attributed to the signals of Ag 3d_5/2_ and Ag 3d_3/2_, respectively, consistent with the reported results^24^. It indicates that the Ag core has no oxidization during the hydrothemal process due to the protection of carbon shell. This is very important for its SERS activity. However, after 365 days ageing, the XPS peaks of Ag element was a bit shifted to lower energy positions, which can be attributed to surface oxidation of Ag nanocrystal. Nevertheless, the content of Ag_2_O is too low to influence on the SERS property as described later. In Fig. 3c, the peak position at 284.8 eV well matches the C1s binding energy, evidencing the existence of carbon coating on Ag NCs. By comparing the two C 1 s XPS spectra, it can be concluded that the carbon shell is rather stable after 365 days storage in water, as the shape of C 1 s peak has no much change. This carbon layer is also characterized by electron energy loss spectroscopy (EELs) as shown in Figure S5, respectively. Further XPS experiment has been made to characterize the oxygen element in our samples 1 day and 365 days after synthesis, respectively, as shown in Fig. 3d. It reveals that the peak at 531.1 eV has no shift after one year ageing. It means that the detected oxygen by XPS could be from the Si substrate instead of Ag@C NCs. The micrograph of our sample in the XPS chamber is shown in Figure S6.

### SERS spectra

**Figure 4:**
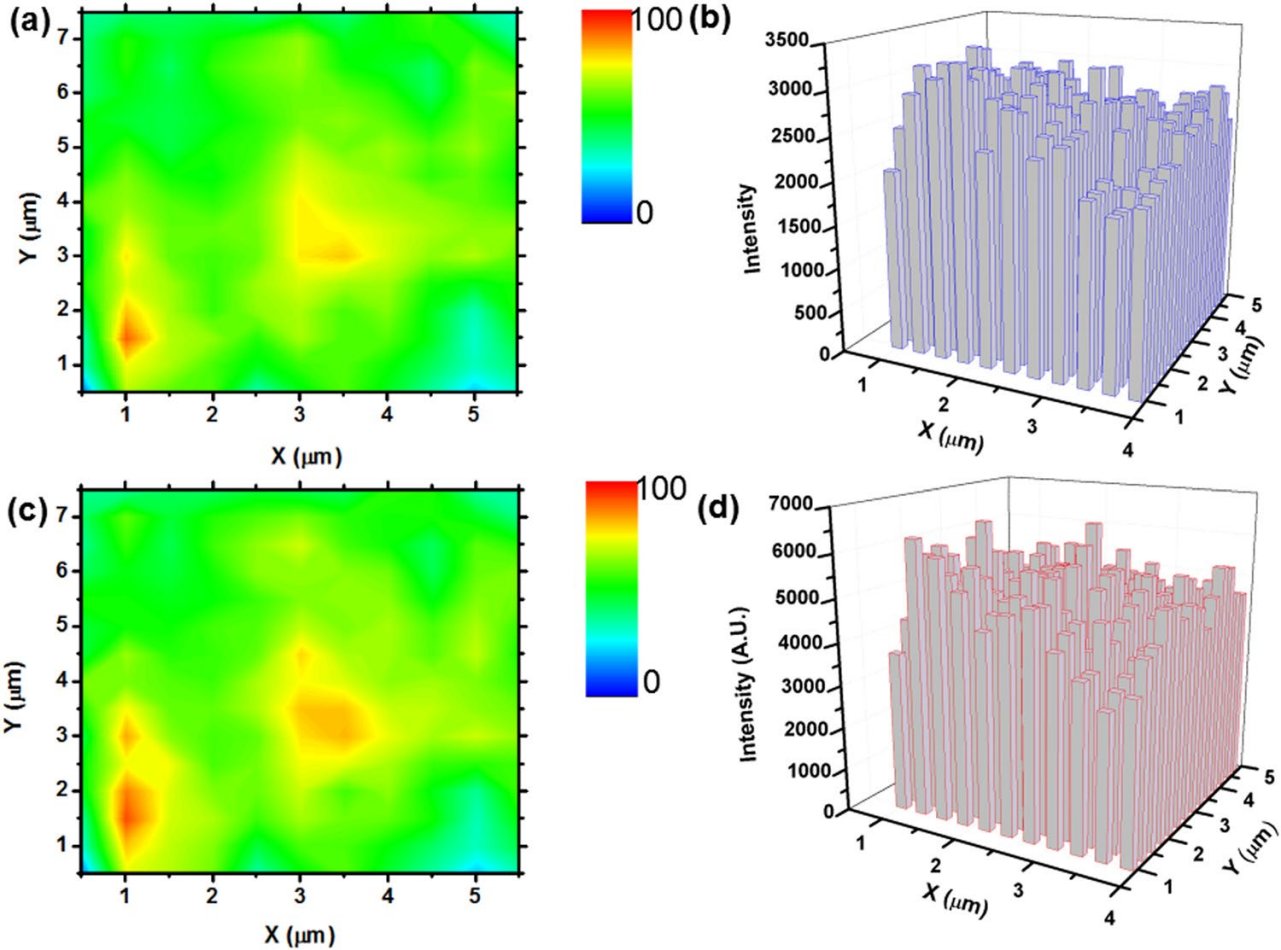
(**a**) and (**c**) SERS intensity maps at 1580 cm^−1^ and 1080 cm^−1^ of 4-MBA on the Ag@C nanocubes surface, respectively. (**b**) and (**d**) the intensity of the peaks in the above area spots.

The SERS property of the Ag@C NCs is studied by using the Raman spectrum of bulk 4-MBA molecules Raman as reference (Figure S7). It can be calculated that the EF factor is approximately 1 × 10^7^, and the detailed analysis method can be seen in Supporting Information. The homogeneity of the Ag@C NCs substrate is clearly revealed by the SEM image in Fig. 1b. However, the homogeneity of SERS signal in a large-scale area should be considered as an important issue in the practical application of these Ag@C NCs as SERS substrates. To study the reproducibility of SERS signals, a spot-to-spot Raman mapping spectrum is collected from the surface of Ag@C nanocubes substrate with 6 × 6 μm area and a step size of 0.5 μm. Figures 4a and c show the 4-MBA molecules Raman intensity distribution of main vibration mode at 1580 cm^−1^ and 1080 cm^−1^, respectively. The Raman intensity distribution of the two peaks reflects the good uniformity and reproducibility of SERS signal in a large scale area. To further demonstrate the SERS uniformity by statistical result, the relative standard deviation (RSD) of the Raman intensity was calculated to be 11.9% for 1580 cm^−1^ and 11.8% for 1080 cm^−1^ (Fig. 4b and d). It confirms the uniformity and reproducibility of the SERS substrate, which makes the temperature-dependent SERS experimental result to be more credible.

**Figure 5:**
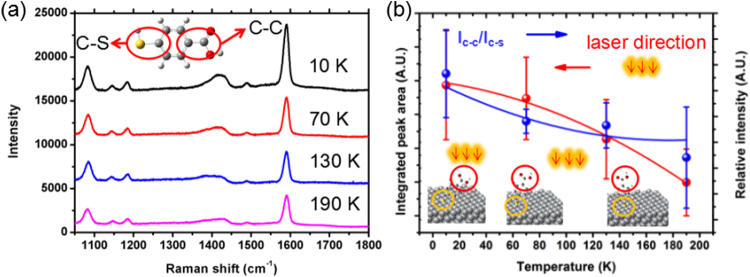
(**a**) SERS spectra of Ag@C NCs at 10 K, 70 K, 130 K and 190 K, respectively. (**b**) The relationship between the SERS integrated peak area of band at 1580 cm^−1^ depending on temperature which is represented by red line (left) and the relative intensity ratio of I_C-C_/I_C-S_ at 1580 cm^−1^ and 1080 cm^−1^ as a function of temperature which is represented by blue line (right). The average relative integrated peak area was recorded from eight measurements on the substrate.

Figure 5a shows the SERS spectra of 4-MBA molecules adsorbed on Ag@C NCs at 10 K, 70 K, 130 K and 190 K, respectively. According to the absorption spectrum of Ag@C NCs in Supporting Information, the SPR peak is in the visible region near 500 nm in wavelength, with the excitation laser source of 488 nm in wavelength (Figure S8). In SERS spectra, the band at 1580 cm^−1^ is assigned to the v_12_ aromatic ring breathing mode and the band at 1080 cm^−1^ is attributed to the v_8a_ C-S stretching mode. The band at 1180 cm^−1^ corresponds to the C-H deformation mode and the weak band at 1410 cm^−1^ is the v(COO-) stretching mode^35^. Other bands are relatively too weak to be determined. The red curve in Fig. 5b shows the relationship between the SERS integrated peak area of band at 1580 cm^1^ and temperatures ranging from 10 K to 190 K (left). Lang *et al*. had reported the temperature-dependent SERS intensity, which was attributed to the antenna effect^14^. The blue curve in Fig. 5b shows the variation of intensity ratio of I_C-C_/I_C-S_ for peaks at 1580 cm^1^ and 1080 cm^1^ against the temperature (right). The relative intensity ratio of I_C-C_/I_C-S_ nonlinearly decreases with increasing the temperature. The lower the intensity ratio of I_C-C_/I_C-S_, the stronger tendency of vertical alignment of adsorbed molecules onto the Ag@C NC substrate^11,36–38^. According to the electromagnetic enhancement mechanism, the SERS signal is inversely proportional to the inter-distance between the vibrating bonds of anchored molecules and the atomic surface of Ag cube. The SERS intensity of C-S stretching should be weak for aromatic rings lying on the Ag@C cube; otherwise for a normal orientation it should be squarely strong. Similar results were obtained by using low-power laser excitation to repeat this experiment, as shown in Figure S9. Our investigation indicates that adsorbed 4-MBA molecules may prefer a flat orientation to the Ag@C NCs surface with decreasing temperature, according to the plot of C-S stretch Raman intensity against temperature. To verify our conclusion, the excited laser of 532 nm at room temperature was used in the parallel experiment to implement this experiment (Figure S10 and Figure S11).

## Discussion Section

**Figure 6:**
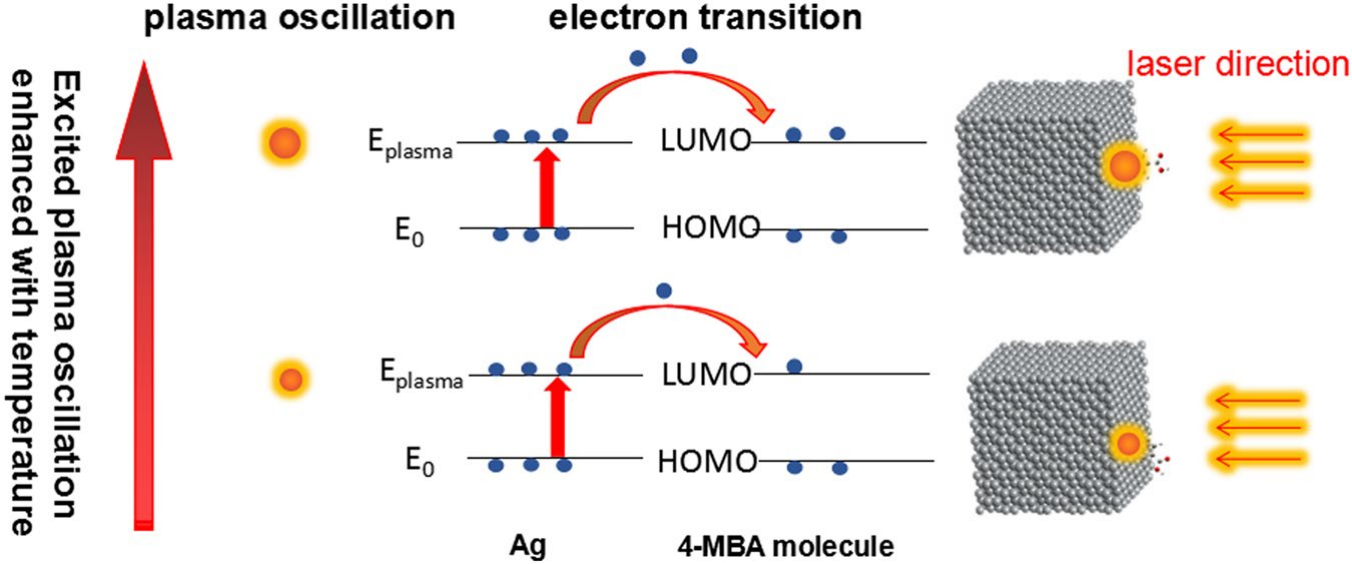
Schematic illustration for the electronic transition processing between the Ag@C NC and adsorbed tip molecule with decreasing temperature.

Figure 6 illustrates the schematic diagram of the possible 4-MBA molecule orientation on Ag@C NCs surface at low and high temperatures. The excited electron can be transferred from the surface plasmon (E_plasmon_) of Ag NC to the LUMO energy level of 4-MBA molecule when under laser illumination. When the temperature is decreased, the number of electrons transferred into the LUMO energy level of target molecule would be less^39^, due to the enhanced recombination between electrons and ions with decreasing the plasmon oscillation^40^. In a strongly acidic environment, C-C bonds of 4-MBA molecules are mainly in the protonated form which is related to the low temperature effect. In our environment, the 4-MBA molecule is adsorbed onto the silver surface through the sulfur atom and the C-C bond may adopt a more vertical orientation with respect to the surface, which suggests that the increase in the C-S band intensity is related to a less acidic ion solution^34^. For further study we changed the pH value of aqueous solution, and the experimental result is shown in Figure S12. The lower the pH value, the higher the intensity ratio of C-C and C-S bands in Raman spectra, and the less electrons transferred from Ag into 4-MBA molecules due to the increased H^+^ around the Ag@C cube to combine with photo-excited electrons. As a result, adsorbed molecules tend to be in flat orientation in acidic solution to meet the minimum energy requirement. The low pH effect is analogous to the low temperature effect, as both inhibit electrons transfer from Ag to 4-MBA molecules.

**Figure 7:**
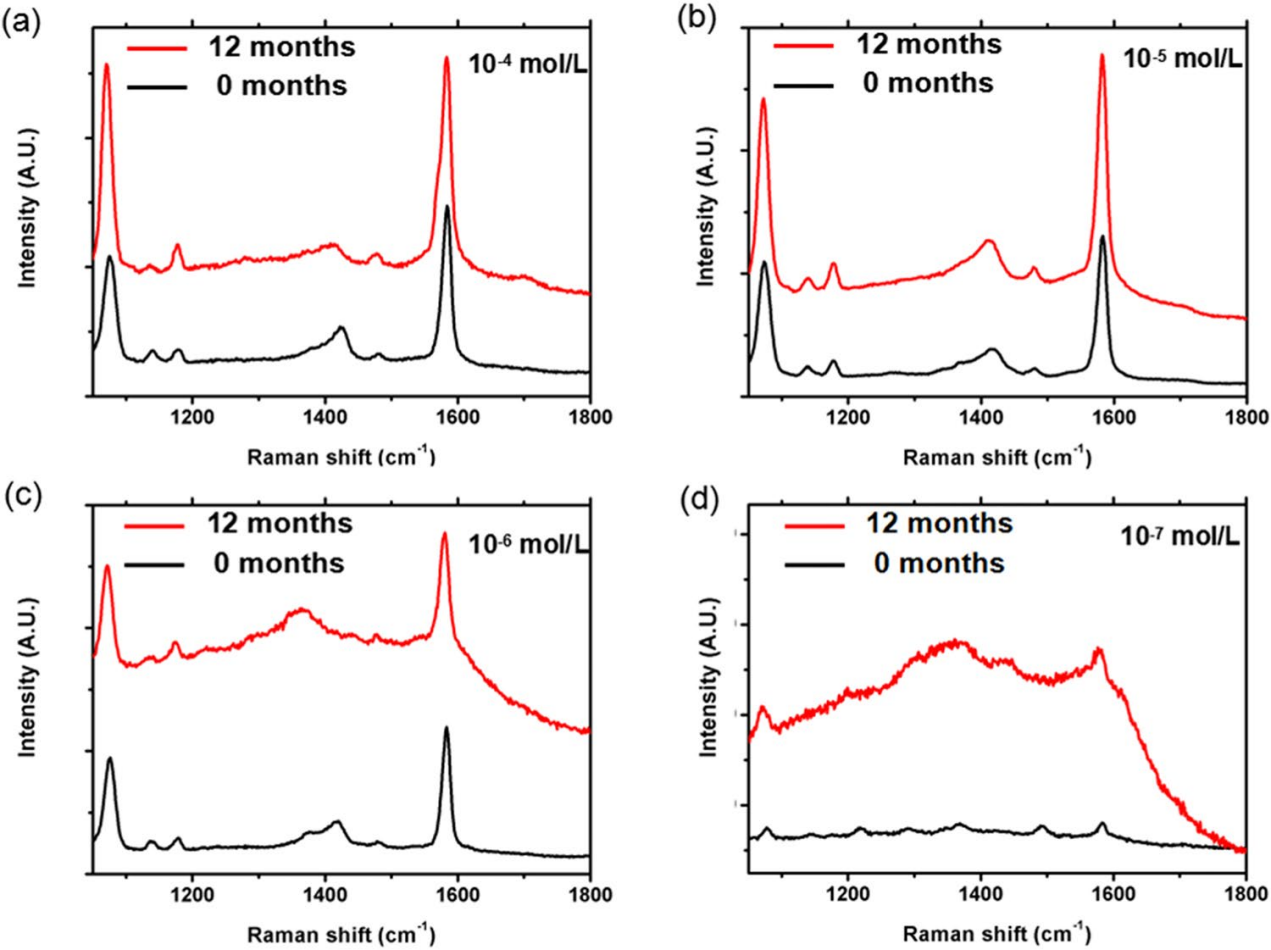
The effect of shelf life on the SERS enhancement: (**a**) 4-MBA(10^−4^ M)/Ag@C NCs; (**b**) 4-MBA(10^−5^ M)/Ag@C NCs; (**c**) 4-MBA(10^−6^ M)/Ag@C NCs; (**d**) 4-MBA(10^−7^ M)/Ag@C NCs.

Recently, core-shell nanostructures^41–44^ have been used to enhance the stability of the noble metal nanostructure^45–47^. However, the problem of Ag core-shell nanostructures is their stability in aqueous solution, because those reported Ag-shell nanostructures usually exhibit a relatively short shelf life^24^.To investigate the stability of our Ag@C NCs prepared by a hydrothermal method, we stored our sample in water solution at room temperature for 12 months and repeated XPS and SERS measurements. Figure 7 shows the SERS spectra of 4-MBA molecules on the surface of Ag@C NCs, with the detection limit of 1 × 10^−7^ mol/L, measured 1 day and 12 months after synthesis. It can be seen in Fig. 7 that the SERS signals remained stable at concentrations of 10^−4^, 10^−5^, 10^−6^ and 10^−7^ mol/L 4-MBA solution, respectively, after one-year storage. The excellent stability of our Ag@C NCs samples against long-term ageing can be attributed to the thin carbon coating. This type of Ag@C core-shell structures may have the potential as a high SERS-active and stable substrate for many bio-sensor applications.

In summary, we have synthesized uniform Ag@C NCs with side length of ~60 nm by a simple hydrothermal approach. The structure and optical properties of Ag@C NCs were characterized using temperature-dependent SERS spectra. The tip molecule orientation on Ag@C substrates was systemically investigated by SERS spectra collected at different temperatures ranging from 10 K to 190 K. It was found that adsorbed 4-MBA molecules on the Ag@C NCs surface could lay down in a flat orientation with decreasing the temperature. Moreover, we studied the stability of Ag@C NCs stored in water at room temperature for over one year. The structure and SERS activity of Ag@C NCs were well maintained after 12 months ageing. Taking the high SERS activity and stability into account, our Ag@C NCs have the potential for further applications in various fields.

## Methods Section

### Materials

All raw materials used in the experiment were without further purification, such as AgNO3 (99.8%), glucose (99.5%), ammonia (25%) and CTAB (98%). Ag@C NCs were obtained by one-step hydrothermal method. Typically, AgNO3 aqueous solution (25.5 mg, 2.5 mL), high purity water (8 mL), glucose aqueous solution (31.5 mg, 2.5 mL) and CTAB aqueous solution (52.9 mg, 3 mL) were orderly added to a 25 mL Teflon-lined autoclave by microinjection pump with 30 min, respectively. Subsequently it was stirred for 1 h and then maintained at 120 °C for 8 h. Ag@C NCs were collected by further centrifugal separation and washed several times with water. The morphology of the products was characterized by Hitachi S-4800 SEM and JEOL 2100 F TEM. The crystal structure and chemical composition of the products were characterized by EDS and XPS (ESCALAB250, Thermo VG).

### Low temperature Raman experiment with 4-MBA as the probe molecule

Ag@C NCs in water solution were dropped onto a silicon wafer and dried. Afterward, the substrate was immersed in a 10^−5^ mol/L 4-MBA ethanol solution for 6 h. Then the Si substrate covered with Ag@C NCs was taken out and constantly rinsed by ethanol. After dried in air, the substrate was transferred into a vacuum chamber with pressure of 10^−4^ torr for low temperature SERS measurement. A Horiba Jobin Yvon Raman spectrometer with 488 nm laser (laser spot size ~1.5 *μ*m and beam power 340 *μ*W) was used to measure the Raman spectra. SERS spectra were collected with a 50 × objective lens.

## Electronic supplementary material

Molecular Tilting Alignment on Ag@C Nanocubes Monitored by Temperature-Dependent Surface Enhanced Raman Scattering
