# Supplementary material for: Molecular Tilting Alignment on Ag@C Nanocubes Monitored by Temperature-Dependent Surface Enhanced Raman Scattering
Source: Sci Rep. 2017 Oct 16;7:12865. doi: 10.1038/s41598-017-13022-x (PMC5643508; doi:10.1038/s41598-017-13022-x)
Supplement: Supplementary file 1 — Molecular Tilting Alignment on Ag@C Nanocubes Monitored by Temperature-Dependent Surface Enhanced Raman Scattering [file 41598_2017_13022_MOESM1_ESM.pdf]

Supporting Information

Molecular Tilting Alignment on Ag@C Nanocubes Monitored by Temperature-Dependent Surface Enhanced Raman Scattering

Yinong Wang[1], Yinghui Sun[2], Di Chen[2], Xiaofang Zhang[2], lin Guo[3], Rongming Wang[2,*]

[1] Department of Physics, Beihang University, Beijing 100191, China

[2]Beijing Key Laboratory for Magneto-Photoelectrical Composite and Interface Science, School of Mathematics and Physics, University of Science and Technology Beijing, Beijing 100083, China

[3]School of Chemistry and Environment, Beihang University, Beijing 100191, China

Corresponding authors.Tel.:+8613391571538.

E-mail addresses: rmwang@ustb.edu.cn

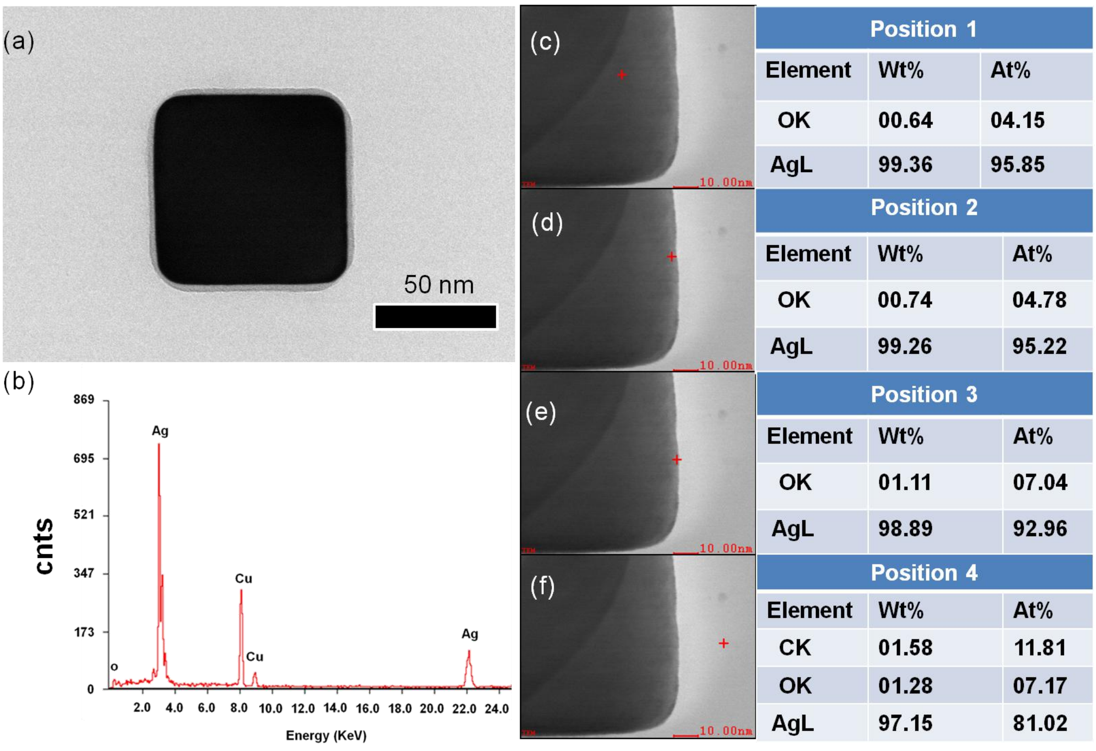

Position 1		
Element	Wt%	At%
OK	00.64	04.15
AgL	99.36	95.85

Position 2		
Element	Wt%	At%
OK	00.74	04.78
AgL	99.26	95.22

Position 3		
Element	Wt%	At%
OK	01.11	07.04
AgL	98.89	92.96

Position 4		
Element	Wt%	At%
CK	01.58	11.81
OK	01.28	07.17
AgL	97.15	81.02

Figure S1. (a). the TEM image silver nanocube. (b). the EDS of silver nanocube. (c), (d), (e) and (f) is the pot-eds of AgK and OK on the silver nanocube. The STEM has been realed the cube shell to be silver material. And it can be further conformed by the pot-eds spectroscopy.

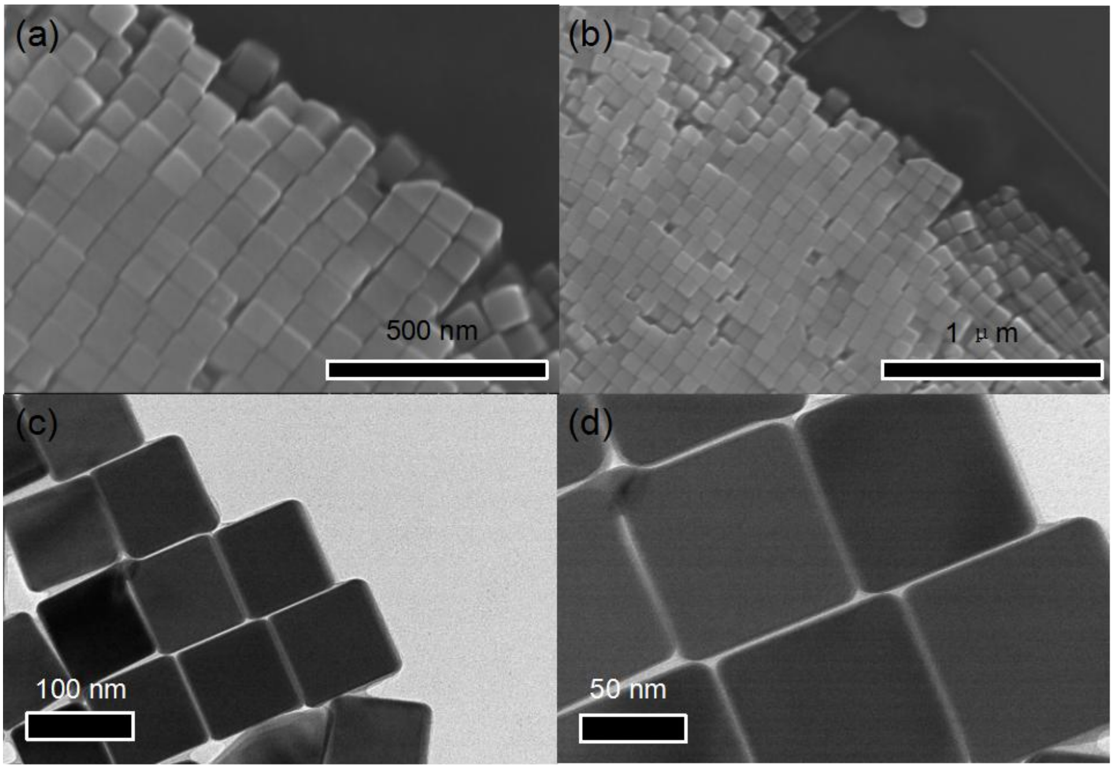

Figure S2. (a) and (b) SEM images of Ag nanocubes. (c) and (d) TEM image of Ag nanocube.

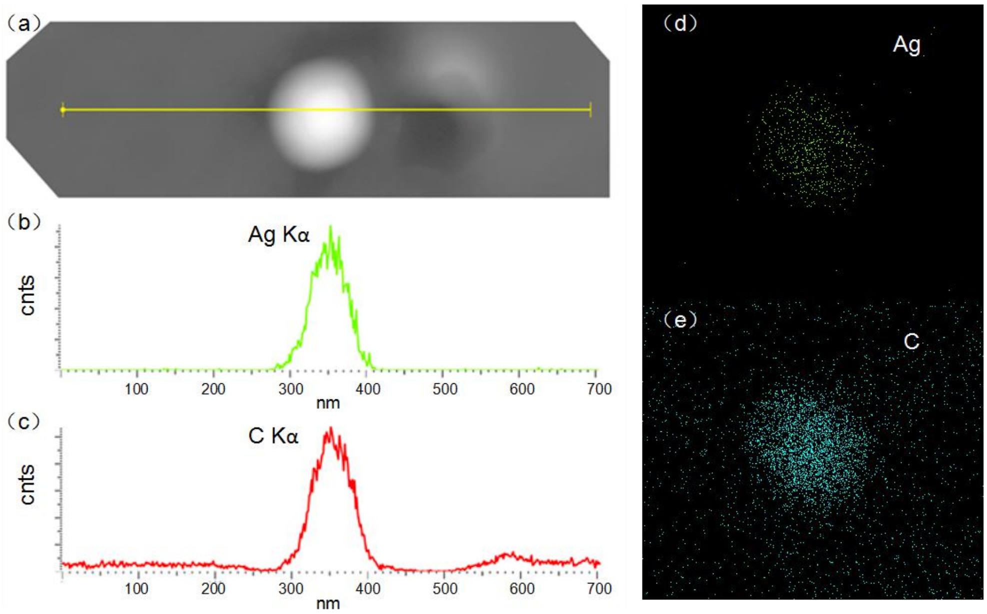

Figure S3. (a) STEM image of the Ag@C NC. (b) and (c) the line STEM-EDS of AgK and CK in the Ag@C NC. (d) STEM-EDS mapping of Ag element in the Ag@C NC. (e) STEM-EDS of C element in the Ag@C NC.

It can be found that Ag@C NCs structure change from corner to curve, which may be come from the bombardment of electron beam.

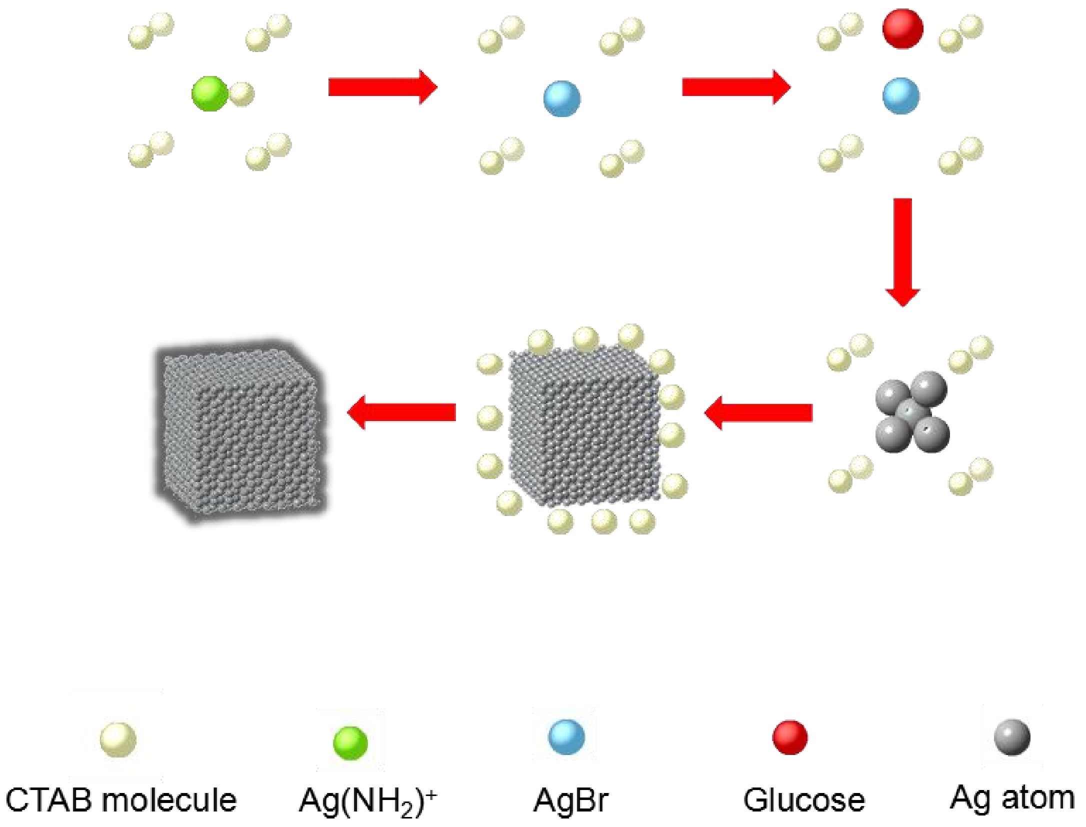

Figure S4. Schematic illustration of Ag@C nanocubes formation process

In the initial step, Ag^{+} was restraint by the CTAB molecules which act as stabilizer agent. And Ag^{+} was deoxidized by the glucose to form Ag nanocubes structure. After that, carbonization with CTAB molecule, the organization of amorphous carbon layer on the Ag nanostructures. In the reaction process, glucose molecules also react as carbon sources to form the carbon shell on the Ag surface.

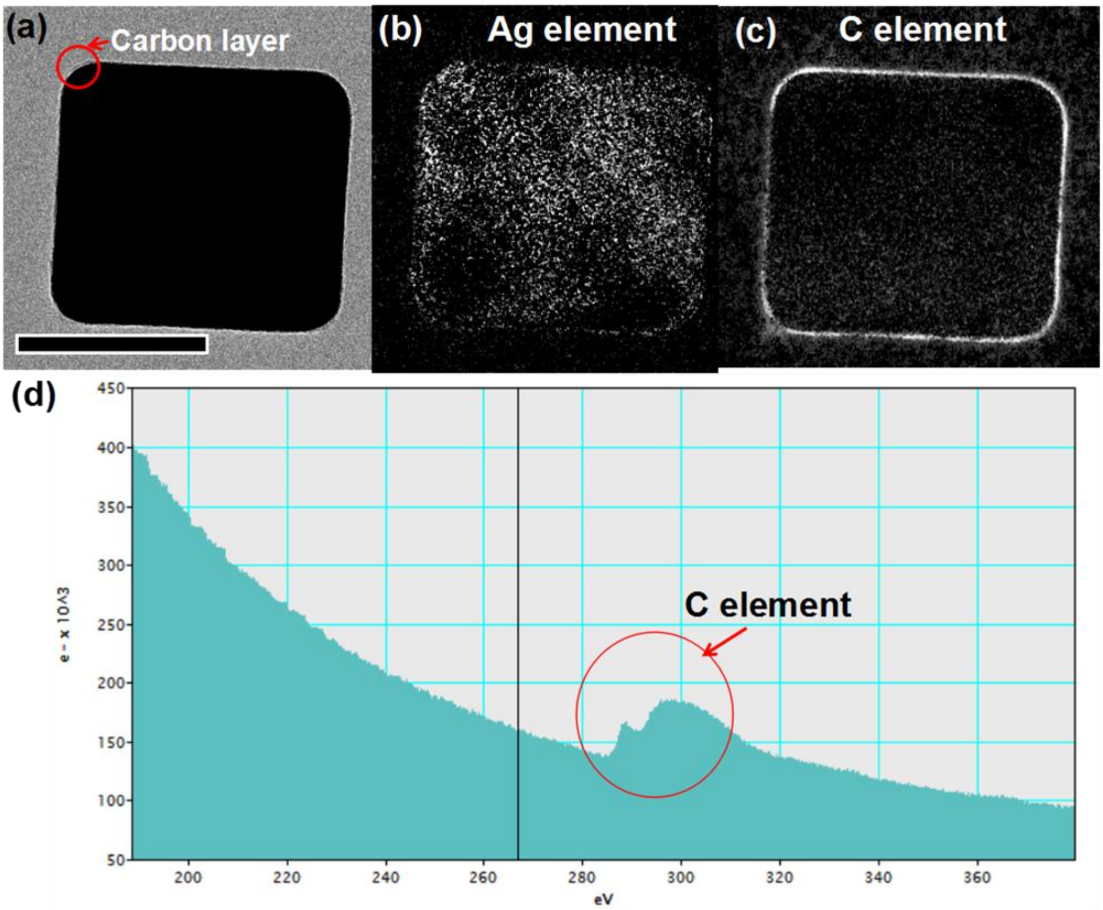

Figure S5. (a) TEM image of the Ag@C NC with scale bar ~50 nm. (b) Energy resolution of Ag in the Ag@C NC. (c) Energy resolution in the C element Ag@C NC. (d) the electron energy loss spectroscopy (EELs) of C element in the Ag@C NC.

4-MBA.

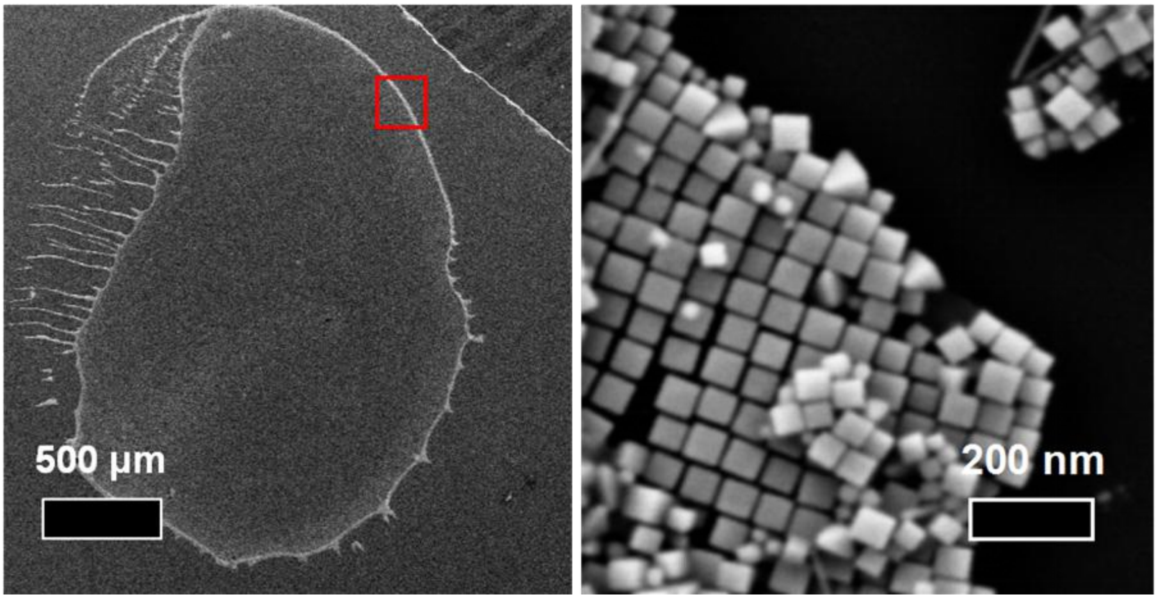

Figure S6. SEM images of Ag@C NCs and the microscopic image (SEM) of the NC distribution area.

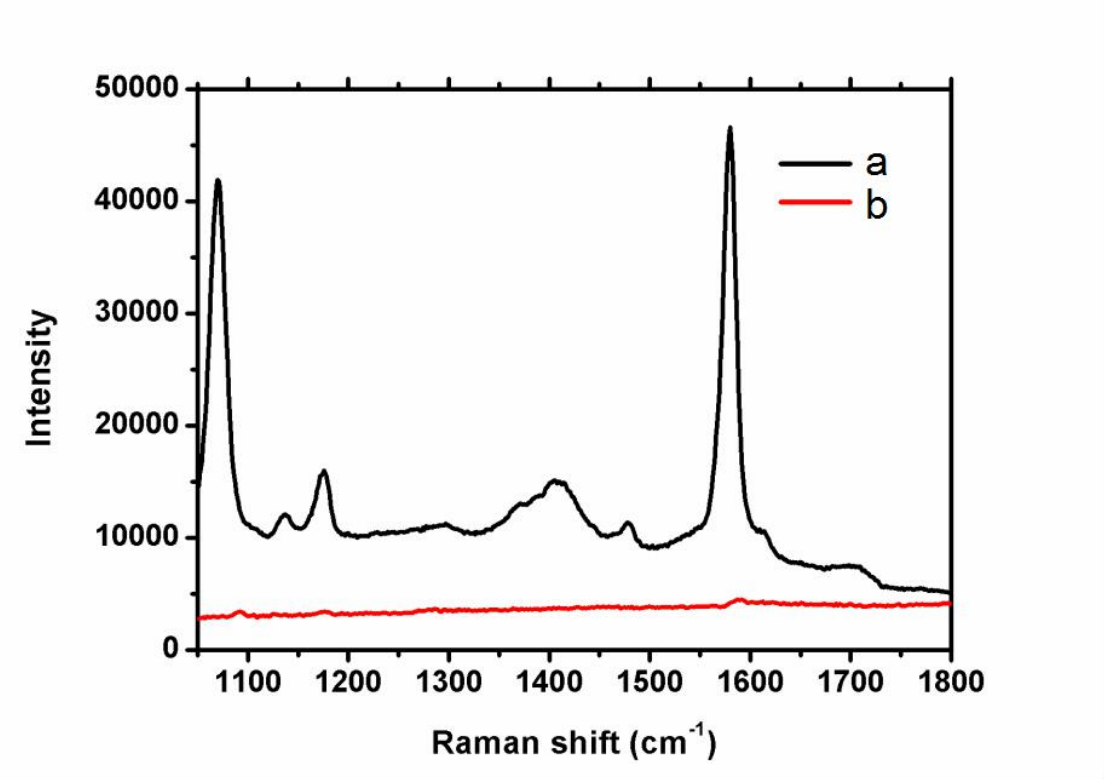

Figure S7 (a) SERS spectrum of 4-MBA on Ag@C NCs. (b) Raman spectrum of solid 4-MBA.

The EF factor was calculated based on the following equations[1]:

G= $(I_{SERs}/N_{SERs})/(I_{bulk}/N_{bulk})$

And the number of tip molecule is represented as:

N_{SERs}=A×N_{sub}×A_{sub}/σ

Where A is the laser irradiation area (~1.5μm), N_{sub} is the Ag@C cube number density (~1.143×10^{-4} nm^{-2}) which is calculated from Figure 1b, A_{sub} is the area of single cube (~60 nm×60 nm), σ is adsorbed area of the single 4-MBA molecule (~0.2 nm^2). It is assumed here that the substrate material surface is fully adsorbed with 4-MBA molecules.

The number of tip molecules contributing Raman signal in the experiment is

N_{bulk}=A×h×n_{bulk}=A×h×N_A×ρ_{bulk}/M_{bulk}

Where A is the laser irradiation area (~1.5μm), h is the laser focusing depth (~15 μm), n_{bulk} is the number of tip molecules per unit volume, ρ_{bulk} is the density of bulk (~10.05 g/cm^3), M_{bulk} is the molecular weight of 4-MBA molecule (~107.9 g/mol), N_A is the Avogadro constant (~6.02×10^{23}).

It can be calculated as：

G= (I_{SERs}/N_{SERs})/(I_{bulk}/N_{bulk})=(I_{SERs}/I_{bulk})×(σ×h×ρ_{bulk}×N_A)/(N_{sub}×A_{sub}×M_{bulk})

So the EF factor G is approximately calculated as 1×10^7.

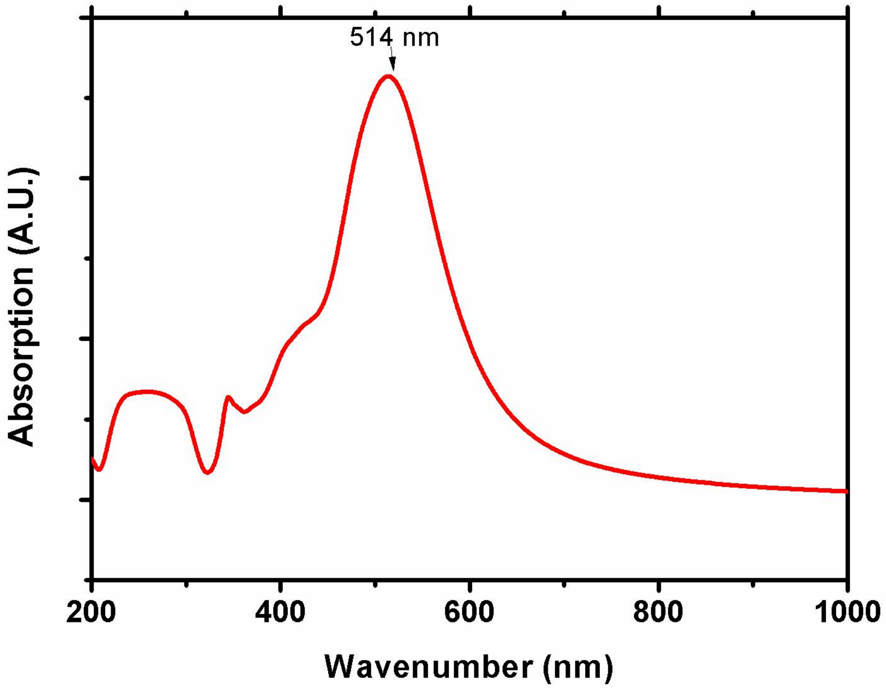

Figure S8. Ag NCs UV−visible absorption spectrum in water solution. And it represents that the absorption peak is at 514 nm, which is accordance with 488nm excited laser.

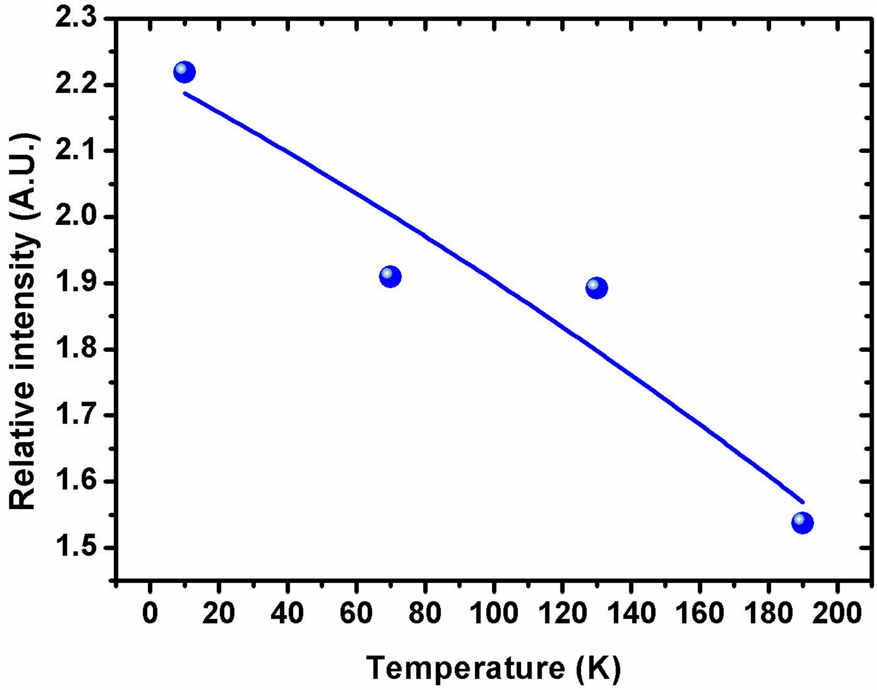

Figure S9. The relative intensity ratio of I_{C-C}/I_{C-S} at 1580 cm^{-1} and 1080 cm^{-1} with 34 μw laser power as a function of temperature

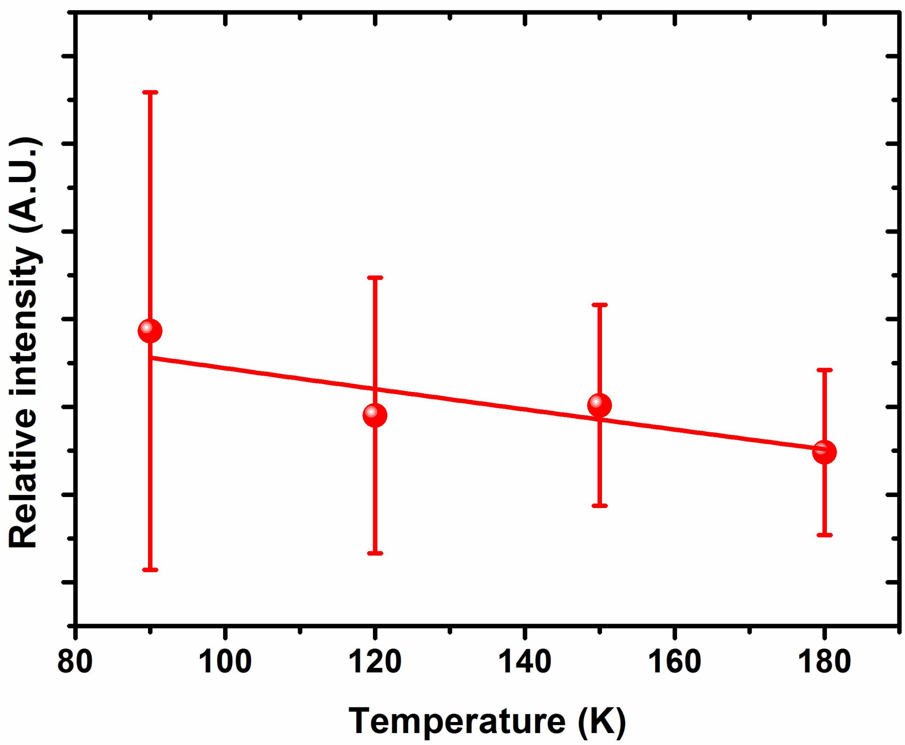

Figure S10. The relative intensity ratio of I_{C-C}/I_{C-S} at 1580 cm^{-1} and 1080 cm^{-1} with 532 nm laser 40 μw power as a function of temperature. The average relative integrated peak area was recorded from eight measurements on the substrate.

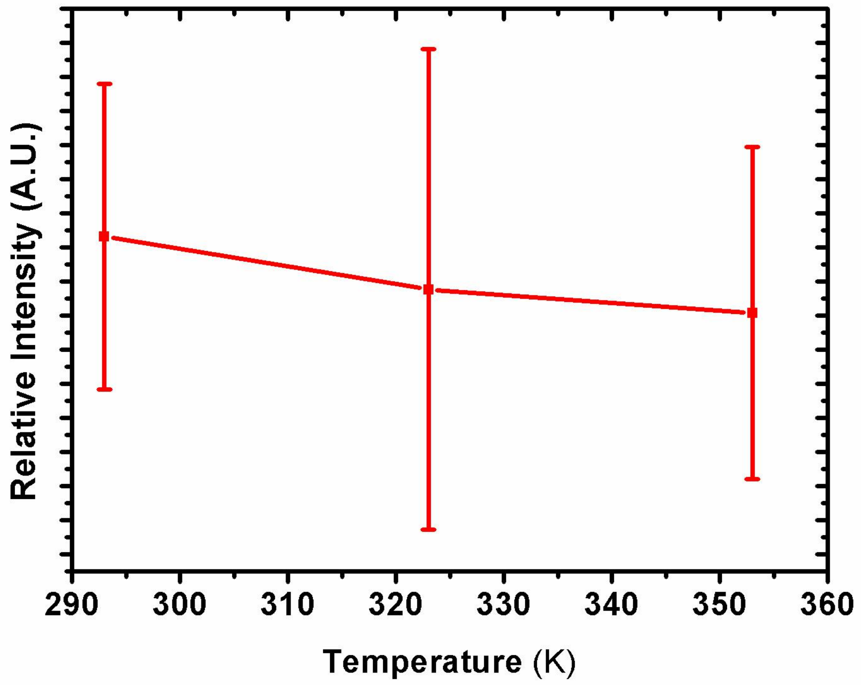

Figure S11. The Ag@C NCs SERS spectra relative intensity ratio of $I_{C\text{-}C}/I_{C\text{-}S}$ at 1580 cm^{-1} and 1080 cm^{-1} as a function of temperature which is represented by the red line at 293 K, 323 K and 353 K, respectively. The average relative integrated peak area was recorded from eight measurements on the substrate.

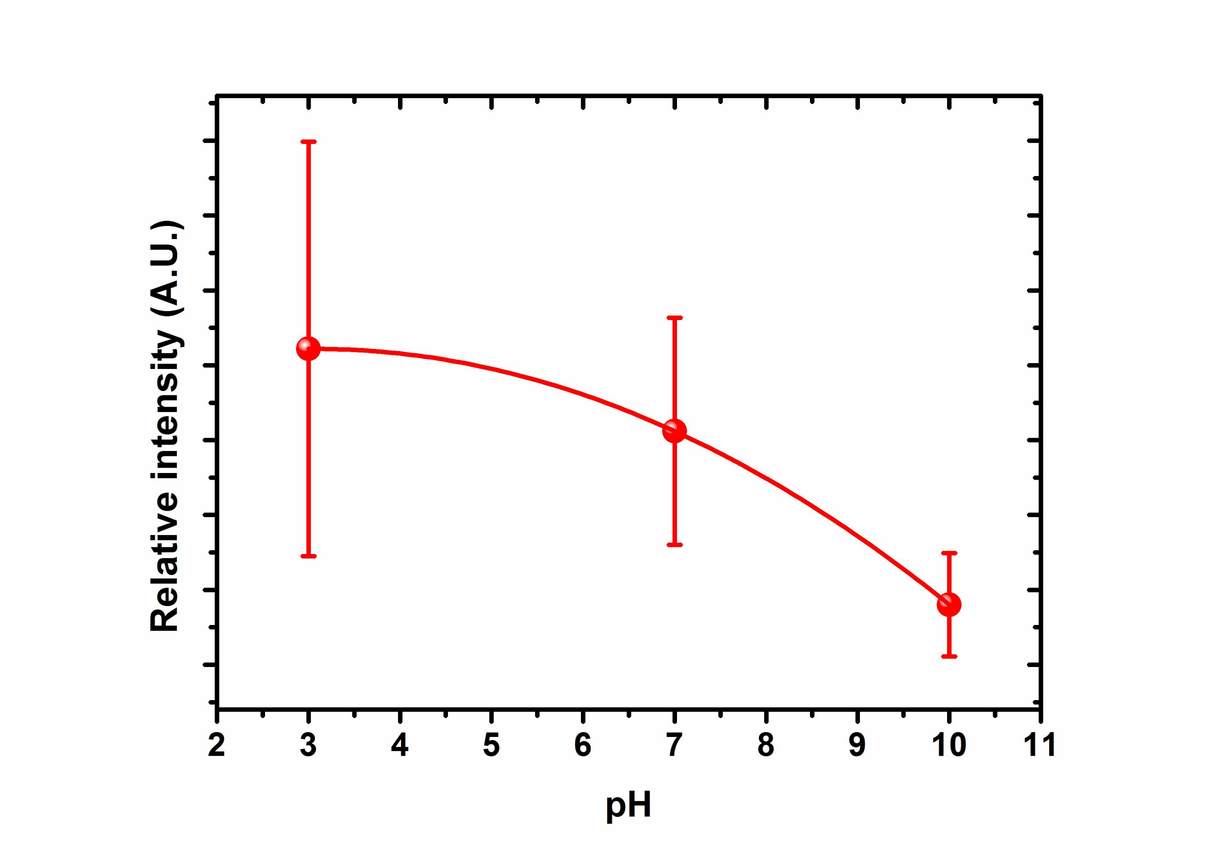

Figure S12. The relative intensity ratio of I_{C-C}/I_{C-S} at 1580 cm^{-1} and 1080 cm-1 at pH=3, 7 and 10.

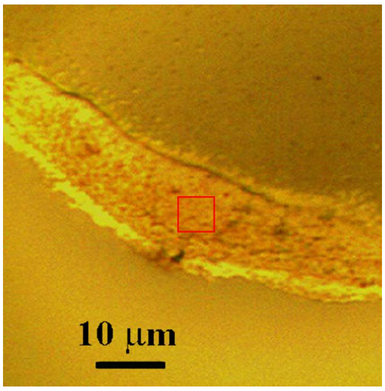

Figure S13. Microscopic image of the NCs distribution

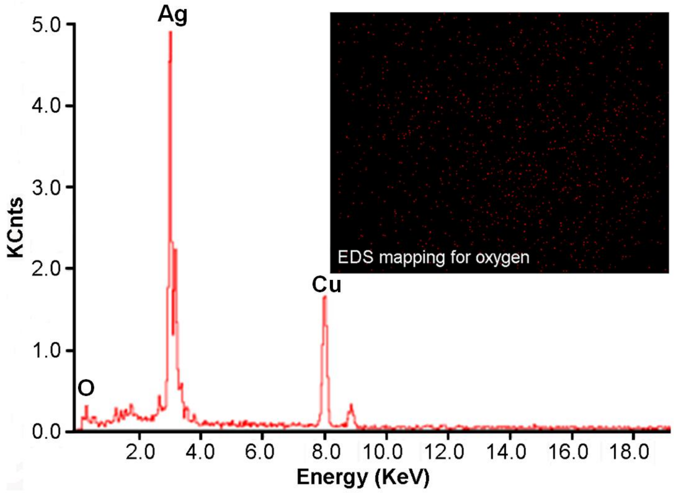

Figure S14. The EDS spectrum of Ag@C NC and STEM-EDS of O K in the Ag@C NC.

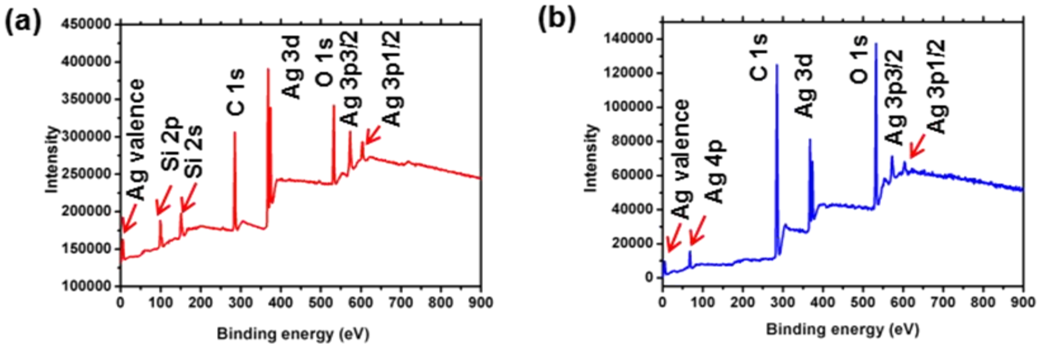

Figure S15. (a) Survey XPS spectrum of Ag NCs on the Si substrate, (b) Survey XPS spectrum of Ag NCs on the conductive adhesives

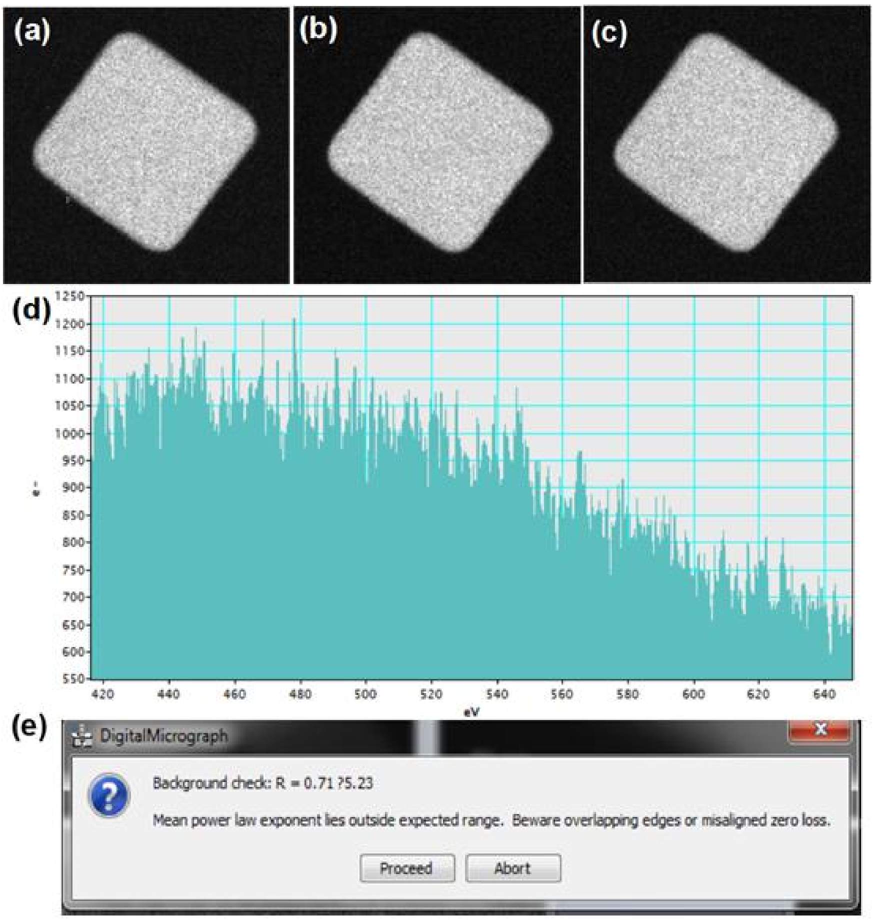

Figure S16. (a), (b) and (c) are the energy resolution of the Ag@C NC with energy range at 515~545 eV (a), 485~515 eV (b) and 455~485 eV (c). (d) the electron energy loss spectroscopy (EELs) of O element in the Ag@C NC. (e) the computing result of the O element energy resolution of the Ag@C NC by the DigitalMicrograph software with three times.

1.Orendorff, C. J.; Gole, A.; Sau, T. K.; Murphy, C. J., Surface-Enhanced Raman Spectroscopy of Self-Assembled Monolayers: Sandwich Architecture and Nanoparticle Shape Dependence. Analytical Chemistry 2005, 77 (10), 3261-3266.
